# Efficacy of Combined Aerobic Exercise and Coaching on Physical Fitness in People With Neuromuscular Diseases: A Randomized Clinical Trial

**DOI:** 10.1212/WNL.0000000000213781

**Published:** 2025-06-04

**Authors:** Sander Oorschot, Merel-Anne Brehm, Annerieke C. van Groenestijn, Tim Veneman, Jos Twisk, Camiel Verhamme, Filip Eftimov, Judith G.M. Jelsma, Vibeke Valkenburg, Esther Kruitwagen, Patrice Tomassen, Heleen van der Wielen, Nicole B.M. Voet, Nicol Cornelia Voermans, Frans Nollet, Eric L. Voorn

**Affiliations:** 1 Department of Rehabilitation Medicine, Amsterdam UMC location University of Amsterdam, the Netherlands;; 2 Amsterdam Movement Sciences, Rehabilitation & Development, the Netherlands;; 3 Department of Epidemiology and Biostatistics, Amsterdam UMC location Vrije Universiteit, the Netherlands;; 4 Department of Neurology and Clinical Neurophysiology, Amsterdam UMC location University of Amsterdam, the Netherlands;; 5 Department of Public and Occupational Health, Amsterdam UMC location Vrije Universiteit, the Netherlands;; 6 Rehabilitation Center Basalt, Leiden, the Netherlands;; 7 Department of Rehabilitation, University Medical Centre Utrecht, the Netherlands;; 8 Center of Excellence for Rehabilitation Medicine, University Medical Centre Utrecht and De Hoogstraat Rehabilitation, the Netherlands;; 9 Department of Rehabilitation, Physical Therapy Science and Sports, Julius Center for Health Sciences and Primary Care, Utrecht, the Netherlands;; 10 Rehabilitation Center Merem, Almere, the Netherlands;; 11 Rehabilitation Center St Maartenskliniek, Nijmegen, the Netherlands;; 12 Department of Rehabilitation, Donders Institute for Brain, Cognition and Behaviour, Radboud University Medical Center, Nijmegen, the Netherlands;; 13 Klimmendaal, Rehabilitation Center, Arnhem, the Netherlands; and; 14 Department of Neurology, Donders Institute for Brain, Cognition and Behaviour, Radboud University Medical Center, Nijmegen, the Netherlands.

## Abstract

**Background and Objectives:**

The quality of evidence for improving physical fitness in people with neuromuscular diseases (NMDs) through aerobic exercise is low. The aim of this study was to evaluate the efficacy of combined personalized home-based aerobic exercise and coaching on physical fitness in people with NMD, compared with usual care.

**Methods:**

In a multicenter, assessor-blinded, randomized controlled trial, participants with different types of NMD were randomized (1:1 ratio) to a 6-month intervention (personalized home-based aerobic exercise and coaching) or usual care. Assessments were performed at baseline, directly after intervention, and at 6 and 12 months after intervention. The primary outcome was physical fitness, measured as peak oxygen uptake (VO_2peak_) directly after intervention. Secondary outcomes included daily physical activity, quality of life, physical functioning, metabolic syndrome markers, and creatine kinase level. We conducted intention-to-treat linear mixed-model analyses for all outcomes, with the baseline value of the particular outcome as covariate.

**Results:**

Ninety-one participants (median age = 64.0, 60% female) were randomized to the intervention (n = 44) or usual care (n = 47) group. The mean group difference in VO_2peak_ directly after intervention was 2.2 mL/min/kg (95% CI 0.2–4.1, *p* = 0.028) and 1.7 mL/min/kg (95% CI 0.1–3.4, *p* = 0.039) on average over time, in favor of the intervention group. There were no significant between-group differences in the secondary outcomes. Twenty-five and 22 adverse events were reported in the intervention and usual care groups, respectively. Creatine kinase levels remained unchanged.

**Discussion:**

This study provides evidence that combined personalized home-based aerobic exercise and coaching is safe and improves physical fitness in people with NMD, but without evidence of improved physical functioning, daily physical activity, quality of life, or metabolic syndrome markers. This home-based approach has good potential for wider implementation. Future research should explore the association between changes in VO_2peak_ and functional outcomes and strategies to counteract the slightly diminishing long-term intervention effect.

**Trial Registration Information:**

The study was registered in the Netherlands Trial Register (ID: NL7344) on November 5, 2018. The first participant enrolled on September 19, 2018. However, the Ethics Review Committee of the Amsterdam Medical Center approved the study protocol on November 7, 2017. No adjustments were made to the approved study protocol, and the register corresponds one on one with the approved study protocol.

**Classification of Evidence:**

This study provides Class II evidence that a 6-month personalized aerobic exercise program combined with coaching improves maximal aerobic capacity in patients with NMDs without effective cures.

## Introduction

Physical inactivity is common among people with neuromuscular diseases (NMDs) because of symptoms such as muscle weakness, fatigue, and pain.^1,2^ Inactivity reduces physical fitness, which is assumed to have a negative impact on daily functioning and social participation.^1,3^ Therefore, promoting physical activity and, with that, physical fitness, is an important goal of neuromuscular rehabilitation.^4^ In recent years, an increasing number of studies demonstrated the potential of aerobic exercise (mainly hospital-based) to safely improve physical fitness.^5-8^ However, the quality of the evidence is still considered low, mainly because most studies were uncontrolled, were underpowered, or lacked intention-to-treat analyses.^5-7^ As a result, the evidence for aerobic exercise in NMD is still inconclusive, and the need for high-quality randomized controlled trials is paramount.^5,6^

Current exercise studies in NMD have predominantly examined short-term effects.^5,7,9^ However, improvements after aerobic exercise are known to diminish in the longer term without ongoing support from health care professionals.^10^ To preserve the health benefits of aerobic exercise in people with NMD, strategies such as behavioral therapy, community-based exercise, and/or technological support are needed to integrate physical activity into daily life and maintain the gains from exercise in the longer term.^10,11^

We developed the IMproving FItness in NEuromuscular diseases (I'M FINE) intervention, which combines personalized home-based aerobic exercise with motivational interviewing (MI) coaching, focused on attaining and preserving an active lifestyle.^12^ The aim of this nationwide, multicenter, randomized controlled trial was to evaluate the short-term and long-term efficacy of the I'M FINE intervention on physical fitness and several secondary outcomes in people with different types of NMD, compared with usual care.

## Methods

### Study Design

We conducted a multicenter, assessor-blinded, 2-armed, randomized controlled trial (RCT), comparing the 6-month I'M FINE intervention with usual care. Participants were enrolled in the intervention between September 19, 2018, and March 22, 2022. Measurements were conducted at baseline (T0), directly after intervention (T1), and at 6 (T2) and 12 months (T3) after intervention. The study was coordinated by Amsterdam UMC, location Academic Medical Center (AMC), where all measurements were performed. The intervention was performed in 2 university hospitals, AMC (Amsterdam) and University Medical Center Utrecht (Utrecht), and 4 Dutch rehabilitation centers: Klimmendaal (Arnhem), Sint Maartenskliniek (Nijmegen), Merem (Almere and Hilversum), and Basalt (Leiden). The full protocol, including a detailed description of the intervention, has been published previously.^12^

### Participants

Participants were recruited from the participating hospitals and rehabilitation centers, through several Dutch members of the European Reference Network of Neuromuscular Disorders and through the national NMD patient organization. Potentially eligible individuals received an information letter. Subsequently, physical fitness and activity levels were discussed during a telephone call with the primary investigator (S.O.). Eligible individuals who were willing to participate were invited to a screening visit. After providing informed consent and being screened by a rehabilitation physician, individuals underwent the baseline assessment (T0) to confirm definitive eligibility for inclusion. Inclusion criteria were as follows: diagnosed with a NMD without effective drug therapy, motivated to improve reduced physical fitness, and aged 18 years or older. We included individuals with different types of NMD, because we hypothesized that common mechanisms underlie reduced physical fitness in different types of NMD, with a focus on post-polio syndrome (PPS), Charcot-Marie-Tooth disease (CMT), and noninflammatory myopathies (including muscular dystrophies and congenital myopathies). We excluded individuals with a contraindication for physical activity according to the American College of Sports Medicine (ACSM) guidelines,^13^ individuals who were unable to follow verbal or written instructions, patients with insufficient competence in the Dutch language, and individuals who had already engaged in an exercise program (i.e., planned, structured, and repetitive physical activity performed at sufficient intensity to improve or maintain physical fitness) for more than 4 weeks in the previous 6 months.

### Randomization and Blinding

After the baseline assessment and study enrollment, participants were randomized in a 1:1 ratio to the I'M FINE program (intervention group) or usual care (control group), using a computer-generated randomization scheme (Castor EDC, Amsterdam, the Netherlands) with random blocks of sequences with variable block sizes of 2 and 4. We stratified for diagnosis (PPS, CMT, other NMDs) and treatment center. The project leader (E.V.), who was not involved in the outcome assessments, performed the randomization. The outcome assessors (S.O. and T.V.) were blinded to group allocation. Participants could not be blinded to group allocation but were instructed not to reveal their group allocation to the outcome assessors.

### Standard Protocol Approvals, Registrations, and Patient Consents

The Medical Ethics Committee of Amsterdam UMC, location AMC, approved the study protocol (NL62104.018.17), and all participating centers granted approval to participate. All participants provided written informed consent. The study was registered in the Netherlands Trial Register (ID: NL7344). The study protocol and statistical analysis plan have been published.^12^

### Intervention

The personalized aerobic exercise program consisted of a 16-week, home-based, polarized program on a stationary ergometer, with approximately 75% of the total training volume performed at low intensities and approximately 25% performed at high intensities. The feasibility of this aerobic exercise program was assessed in a previous pilot study.^14^ Physical therapists trained for this study supervised the aerobic exercise program in 6 face-to-face sessions and 3 telephone sessions. Each week, 2 low-intensity sessions below the first ventilatory threshold (VT1) and 1 high-intensity session above the VT1 were performed. The VT1 is a physiologic marker that distinguishes between low and high exercise intensities and is widely used to determine personalized target intensity zones for training.^15^ Training sessions consisted of multiple exercise bouts interspersed by recovery bouts. The exercise bouts were progressively increased in duration (details can be found in the study protocol^12^). Target heart rate ranges were based on the VT1 and ranged between 80% and 100% of VT1 during the low-intensity sessions and between 105% and 115% of VT1 during the high-intensity sessions. VT1 was determined from a maximal exercise test at baseline and reassessed after 8 weeks of training.^16^ When VT1 could not be assessed, or participants were unable to exercise guided by heart rate (e.g., in case of beta-blocking agents), intensity was prescribed based on ratings of perceived exertion, using the 6 to 20 Borg scale.^17^ Participants were provided with a heart rate monitor chest strap (Polar H10; Polar Electro, Kempele, Finland) and the ReVi app (Amsterdam UMC, Amsterdam, the Netherlands). The ReVi app was specifically designed for I'M FINE and programmed with the participant's target heart rate ranges during the training sessions.^18^ Real-time auditory feedback was provided to support participants to maintain their heart rate within the target range.

Alongside the aerobic exercise program, participants received coaching based on MI. MI is a participant-centered communication technique that helps participants change their behaviors by exploring and resolving their ambivalence toward behavior change in a nonconfrontational style.^19^ The coaching consisted of 8 individual face-to-face sessions and 3 telephone sessions over 6 months. Sessions focused on identification of individual beliefs and aims toward a physically active lifestyle. The core elements of the coaching program were education about physical fitness in NMD, goal setting, personal coaching, and feedback on daily physical activity (for which participants were provided with a FITBIT Flex [Fitbit Inc., San Francisco, CA]). All sessions included homework assignments, and a close relative attended several sessions to discuss ongoing support and options for transitioning from ergometer training to daily activities or sports participation. MI-trained occupational therapists or movement teachers supervised the coaching program. Before the start of the study, an experienced MI assessor (J.J.) trained these therapists and teachers by providing feedback on a test audio recording.

Attendance rates (number of sessions attended) for aerobic exercise and coaching were assessed using the ReVi dashboard and therapist logbooks.

Participants in the control group received usual care and were instructed to continue with their activities as usual. All participants were allowed to use or be prescribed assistive devices, orthoses, regular physical therapy, and/or medication and were not restricted in their activities.

### Outcome Measures

We collected primary and secondary outcomes, as well as adverse events (i.e., any undesirable experience occurring to a participant during the study, whether considered related to the intervention or measurements), at baseline (T0), directly after intervention (T1), and at 6 months (T2) and 12 months (T3) after intervention. Outcomes were entered into a Castor EDC database. At baseline, we also collected demographic and clinical characteristics (type of NMD, sex, age, ethnicity, marital status, education level, work status, body mass index, manual muscle testing [MMT] sum scores,^20^ walking aids, self-reported functional ambulation,^21^ and spirometry values).

The primary outcome was the difference in peak oxygen uptake (VO_2peak_) between the groups directly after intervention. We measured VO_2peak_, a valid measure of the maximal aerobic capacity in NMD,^22^ during a maximal cardiopulmonary exercise test (CPET) on a bicycle or arm ergometer (Lode Excalibur, Groningen, the Netherlands) using a breath-by-breath gas-analysis system (MasterScreen CPX; CareFusion, Hoechberg, Germany). Participants followed a ramp protocol with continuous workload increments of 5–20 W/min, depending on the participants' physical fitness level. The test was stopped if the participant reached a VO_2_ plateau, exhaustion, or any of the ACSM stopping criteria,^13^ or if the pedal frequency dropped below 50 revolutions per minute. The CPET data were analyzed using a self-developed Matlab script (The MathWorks Inc., Natick, MA). Because CPET data can be affected by various measurement artifacts, we removed outliers in the breath-by-breath respiratory gas exchange data (i.e., >2 SD from the 10-breath mean) and determined VO_2peak_ as the highest 15-second moving average achieved during CPET.^22,23^

Secondary outcomes included daily physical activity (measured as time per day spent in moderate-intensity and vigorous-intensity activities and daily step count), measured during 7 consecutive days; health-related quality of life (assessed with the Short Form 36-Item [SF-36] Health Survey^24^); perceived physical functioning (ACTIVLIM^25^); muscle strength, quantified as the maximal voluntary torque assessed on a fixed dynamometer (Biodex, Shirley, NY) of the upper extremity muscles (elbow flexors and/or shoulder abductors) or lower extremity muscles (knee extensors and/or plantar flexors), depending on the selected exercise mode; markers of metabolic syndrome (total cholesterol, high-density lipoprotein, low-density lipoprotein, very low–density lipoprotein, triglycerides, glucose, waist circumference) and serum creatine kinase level as a recommended outcome to monitor safety in exercise intervention studies in NMD; self-efficacy (Self Efficacy for Physical Activity Scale^26^); and physical capacity (total distance walked and oxygen consumption during a 6-minute walk or push test at self-selected, comfortable speed).

### Sample Size

Our study was powered to detect a difference in VO_2peak_ between the intervention and control groups both in the short term (T1, primary outcome) and in the longer term (T2). Because the change in VO_2peak_ at T1 was expected to be slightly larger, we used the difference in VO_2peak_ at T2 for the sample size calculation. Based on previous studies of exercise programs in NMD, we expected a difference in VO_2peak_ at T2 between the intervention and control groups of 2.5 mL/min/kg (10%).^27-29^ Based on a 1:1 group allocation, a SD in VO_2peak_ of 4.7 mL/min/kg,^6^ and a 2-sided α of 0.05, we needed 76 participants per group to achieve 90% power. As the baseline value of each outcome served as a covariate in all separate analyses, a correction was made for the correlation coefficient between baseline and follow-up scores.^30^ The correlation coefficient for VO_2peak_ was found to be 0.71 in a previous RCT by our group.^31^ This resulted in 38 participants per group (76 × [1 − *r*^2^]), for a total of 76 participants. Because a maximum dropout rate of 15% was expected based on previous studies,^31,32^ we aimed to recruit 90 participants.

### Statistical Analysis

After checking the normal distribution of the residuals, we conducted linear mixed-model analyses, with intention to treat, to analyze the intervention effects of all particular outcomes (primary and secondary) on average over time (i.e., from T1 to T3) and at the specific measurement occasions (i.e., T1, T2, and T3). To analyze the intervention effects at the different follow-up measurements, time (treated as a categorical variable and represented by dummy variables) and the interaction between “group” and “time” were added to the model. In all models, we adjusted for the baseline value of the particular outcome and we included a random intercept at the participant level to adjust for the dependency of the repeated observations within the participant, and, in addition, a random intercept for treatment center, to account for the partial clustering of participants within centers. We tested for possible confounding of all baseline characteristics (Table 1) on the primary outcome in a sensitivity analysis. Only those potential confounders that changed the intervention effect by at least 10% were added to the model. In addition, to examine the possible effect modification of diagnosis, an interaction between “group” and “diagnosis” was added to the model of the primary outcome. If this interaction was statistically significant, we performed subgroup analyses for the different diagnoses. All statistical analyses were performed using IBM SPSS Statistics for Windows, version 28 (IBM Corp, Armonk, NY).

**Table 1:** Baseline Characteristics of the Participants

	Control (n = 47)	Intervention (n = 44)
Diagnosis		
Post-polio syndrome	14 (30%)	12 (27%)
Charcot-Marie-Tooth disease	20 (43%)	20 (46%)
Type 1	6/20 (30%)	12/20 (60%)
Type 2	10/20 (50%)	5/20 (25%)
Type 5	2/20 (10%)	—
Type X	2/20 (10%)	1/20 (5%)
Unknown	—	2/20 (10%)
Other	13 (28%)	12 (27%)
Congenital myopathy	4/13 (31%)	3/12 (25%)
Limb-girdle muscular dystrophy	3/13 (23%)	2/12 (17%)
Myotonic dystrophy	2/13 (15%)	3/12 (25%)
Becker muscular dystrophy	—	1/12 (8%)
Ullrich congenital muscular dystrophy	—	1/12 (8%)
Oculopharyngeal muscular dystrophy	1/13 (8%)	—
Inclusion body myositis	2/13 (15%)	1/12 (8%)
Myasthenia gravis	1/13 (8%)	—
Chronic idiopathic axonal polyneuropathy	—	1/12 (8%)
Sociodemographic characteristics		
Sex, female	31 (66%)	24 (55%)
Age, y	65 (51–68)	64 (48–68)
Caucasian ethnicity	43 (92%)	38 (86%)
Married or partner	31 (66%)	26 (59%)
College or university degree	27 (58%)	32 (73%)
>20 h/wk paid job	16 (34%)	17 (39%)
Body mass index (kg/m^2^)	26.8 (4.9)	25.3 (4.1)
Manual muscle testing sum score in lower extremity (0–80)	74.0 (61.5–79.0)	73.8 (63.8–76.0)
Use of walking aids	13 (28%)	14 (32%)
Adapted footwear	28 (60%)	19 (43%)
Lower extremity orthosis	10 (21%)	14 (32%)
Self-reported functional ambulation		
In and around the house	9 (20%)	6 (14%)
Seldom further than 1 km	11 (24%)	12 (27%)
Regurlarly further than 1 km	26 (57%)	26 (59%)
Beta-blocker use	5 (11%)	5 (11%)
Spirometry values		
Forced vital capacity (L)	3.6 (0.9)	3.7 (1.0)
Forced expiratory volume in 1 s (L)	2.8 (0.7)	2.8 (0.8)
Maximal voluntary ventilation (L/min)	85.9 (28.5)	96.0 (34.9)
Peak oxygen uptake (mL/min/kg)	20.2 (7.3)	22.3 (6.5)

Data are mean (SD), median (interquartile range), or n (%). Some percentages do not sum up to 100% because of rounding.

### Data Availability

Study protocols and anonymized data will be made available on request.

## Results

### Participants

The flowchart of the study participants is shown in the Figure. Of the 363 patients assessed for eligibility, 91 met the inclusion criteria. The main reason for refusal to participate given by people who were reached (most people in the “other reasons” group could not be reached after sending the invitation letter) was that the trial was too time-consuming. Six participants in the intervention group and 12 participants in the control group were lost to follow-up, mainly for medical reasons (e.g., cancer diagnosis, thyroid problems, and joint concerns). Twenty-two measurement visits were cancelled (13 in the intervention group and 9 in the control group) because of the coronavirus disease 2019 (COVID-19) measures, and 2 visits were missed for a medical reason (broken toe; same participant in the intervention group at 2 measurement occasions). The participants' baseline characteristics are presented in Table 1. The intervention and control groups were well balanced regarding clinical characteristics (e.g., type of NMD, MMT, walking aids, and functional ambulation levels).

**Figure:**
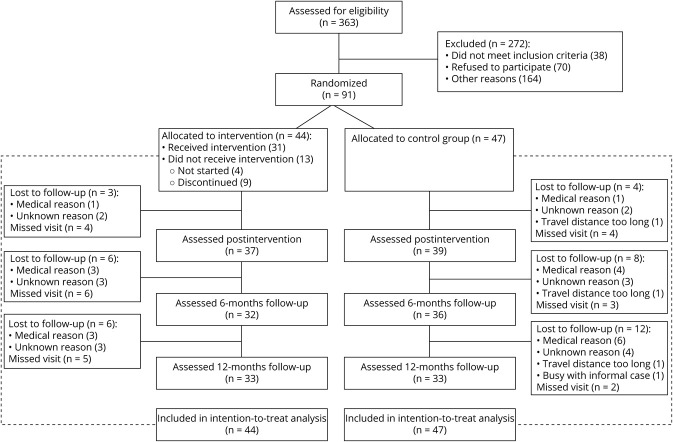
Flowchart of the Study Participants Subtracting the sum of lost to follow-up and missed visits at that measurement occasion from the total number of participants allocated to the intervention or control group gives the number of participants at each measurement occasion.

### Intervention

Most participants were allocated to a university hospital (n = 56). Four participants in the intervention group (9%) did not start the I'M FINE intervention after randomization, because they believed that it was not possible to integrate the intervention into their daily lives (n = 3) or for family reasons (n = 1). The median number of aerobic exercise sessions completed was 41 of 48 (interquartile range [IQR] 23–47), and the median number of coaching sessions completed was 10 of 11 (IQR 8–11).

### Outcomes

The residuals of all outcome variables were normally distributed. Directly after intervention, the mean VO_2peak_ was significantly higher in the intervention group than in the control group (2.2 mL/min/kg, 95% CI 0.2–4.1). On average over time (i.e., from T1 to T3), the mean VO_2peak_ was 1.7 mL/min/kg (95% CI 0.1–3.4), significantly higher in favor of the intervention group. The observed means and intervention effects (based on the linear mixed-model analyses) over time and at the different measurement occasions are given in Table 2. Sex was the only relevant confounder, and adding this variable to the model in the sensitivity analysis resulted in a slightly reduced significant intervention effect of 2.0 mL/min/kg directly after intervention (95% CI 0.0–3.9) in favor of the intervention group. There was no significant interaction between intervention and diagnosis, so no subgroup analyses were performed. Despite 4 secondary outcomes at different measurement occasions, none of the secondary outcomes showed significant intervention effects. The observed means and intervention effects of the secondary outcomes are provided in Tables 3 and 4, respectively. Twenty participants (45%) in the control group and 16 participants (36%) in the intervention group were prescribed new cointerventions (lower limb orthoses, orthopaedic footwear, or physical therapy) during the study period. Owing to COVID-19 measures, previously used sports or physical therapy facilities were closed for longer than 4 weeks for 8 participants in the control group (17%) and 4 participants in the intervention group (9%).

**Table 2:** Observed Means (SD) and Intervention Effects on Peak Oxygen Uptake at the Different Measurement Occasions

	Control		Intervention		Intervention effect	
	N	Observed means	n	Observed means	β (95% CI)	*p* Value
Over time	—	—	—	—	1.7 (0.1 to 3.4)	0.039
After intervention	37	20.5 (7.3)	35	24.8 (8.3)	2.2 (0.2 to 4.1)	0.028
6 mo after intervention	33	22.2 (8.0)	32	24.7 (8.2)	1.6 (−0.4 to 3.6)	0.116
12 mo after intervention	30	20.9 (7.3)	33	23.9 (9.4)	1.4 (−0.6 to 3.5)	0.167

The intervention effects are based on the linear mixed-model analyses.

**Table 3:** Observed Means (SD) or Median [IQR] of the Secondary Outcomes at the Different Measurement Occasions

	Baseline		After intervention		6-mo follow-up		12-mo follow-up	
	N	Mean (SD) or median [IQR]	N	Mean (SD) or median [IQR]	N	Mean (SD) or median [IQR]	N	Mean (SD) or median [IQR]
Steps (daily)								
Intervention	36	4,287 [2,317–5,114]	27	4,877 [3,159–6,970]	30	4,875 [3,868–5,789]	32	4,991 [3,281–5,904]
Control	41	4,634 [3,357–6,697]	32	4,172 [2,646–5,871]	29	4,729 [2,572–5,996]	28	4,673 [2,570–6,685]
Time spent in MVPA (min/daily)								
Intervention	28	91 [20–152]	21	93 [44–112]	26	72 [42–163]	22	117 [67–190]
Control	35	112 [64–188]	25	86 [54–167]	22	106 [62–186]	25	107 [77–159]
SF-36: physical component score (0–100)								
Intervention	44	41.4 (8.5)	40	42.3 (9.5)	37	41.6 (9.8)	37	41.3 (9.5)
Control	46	38.0 (10.4)	42	38.4 (11.4)	42	37.3 (11.1)	38	38.0 (11.5)
SF-36: mental component score (0–100)								
Intervention	44	54 (3.4)	40	53.9 (3.7)	37	54.6 (3.5)	37	54.2 (3.1)
Control	46	54.8 (3.9)	42	54.2 (5.1)	42	55.2 (4.4)	38	55.0 (4.4)
ACTIVLIM (−6.6 to 6.3)								
Intervention	44	2.4 (1.7–3.6)	40	2.6 (1.7–3.5)	37	2.7 (2.1–3.5)	37	2.5 (1.6–3.5)
Control	46	2.1 (1.5–3.1)	42	1.9 (1.4–3.1)	42	2.1 (1.4–2.7)	38	2.1 (1.2–2.9)
Self-efficacy scale (0–25)								
Intervention	44	19.2 (3.1)	40	17.2 (3.7)	37	17.5 (3.6)	37	17.0 (3.7)
Control	46	18.3 (3.7)	42	16.4 (3.7)	42	16.6 (3.0)	38	15.6 (4.5)
MVT musculus quadriceps strongest leg (Nm)								
Intervention	44	106.5 (44.5)	37	107.9 (38.4)	30	114.9 (41.3)	33	113.0 (43.7)
Control	44	98.0 (41.8)	36	96.3 (46.0)	35	98.1 (45.5)	29	97.4 (45.4)
MVT musculus triceps surae strongest leg (Nm)								
Intervention	33	42.7 (26.0)	26	43.7 (24.1)	20	49.7 (27.8)	23	43.2 (24.7)
Control	44	39.3 (18.9)	34	37.5 (19.3)	31	42.5 (23.2)	25	40.2 (21.4)
Cholesterol (mmol/L)								
Intervention	44	5.55 (0.97)	37	5.44 (1.01)	32	5.44 (1.18)	33	5.34 (0.97)
Control	46	5.36 (0.94)	38	5.52 (1.17)	34	5.37 (1.12)	33	5.26 (1.07)
HDL (mmol/L)								
Intervention	44	1.67 (0.39)	37	1.70 (0.44)	32	1.77 (0.39)0	33	1.70 (0.37)
Control	46	1.64 (0.43)	38	1.72 (0.46)	34	1.74 (0.42)	33	1.67 (0.46)
LDL (mmol/L)								
Intervention	44	3.41 (0.86)	37	3.29 (0.93)	32	3.20 (1.17)	33	3.15 (0.86)
Control	46	3.27 (0.79)	38	3.40 (0.97)	34	3.23 (0.87)	33	3.16 (0.90)
VLDL (mmol/L)								
Intervention	44	3.50 (1.00)	37	3.39 (1.11)	32	3.24 (1.11)	33	3.28 (0.92)
Control	46	3.43 (0.90)	38	3.33 (1.06)	34	3.19 (0.73)	33	3.32 (0.98)
Triglycerides (mmol/L)								
Intervention	44	1.04 (0.61)	37	1.01 (0.57)	32	0.99 (0.57)	33	1.08 (0.80)
Control	46	1.02 (0.54)	38	0.99 (0.55)	34	0.90 (0.39)	33	0.97 (0.52)
Glucose (mmol/L)								
Intervention	44	5.4 (0.7)	37	5.3 (0.6)	32	5.4 (0.6)	33	5.2 (0.6)
Control	46	5.4 (0.5)	38	5.5 (0.6)	34	5.4 (0.5)	33	5.6 (0.6)
Waist circumference (cm)								
Intervention	32	94.6 (10.3)	32	94.3 (12.8)	28	93.7 (11.5)	31	95.6 (12.0)
Control	33	96.1 (12.1)	31	92.6 (9.3)	34	93.9 (9.2)	30	93.2 (8.0)
Creatine kinase (U/L)								
Intervention	44	196 (165)	37	245 (234)	32	211 (176)	33	217 (176)
Control	46	199 (451)	38	195 (450)	34	227 (600)	33	286 (958)
Blood pressure: diastolic (mm Hg)								
Intervention	44	126 (13)	35	122 (18)	32	119 (16)	33	120 (18)
Control	47	133 (17)	39	125 (14)	32	125 (15)	29	123 (13)
Blood pressure: systolic (mm Hg)								
Intervention	44	76 (10)	35	75 (12)	32	72 (11)	33	75 (10)
Control	47	78 (8)	39	75 (9)	32	71 (10)	29	74 (10)
WECT: distance (m)								
Intervention	42	371.4 (74.3)	37	385.6 (74.3)	32	400.3 (80.3)	33	391.9 (89.8)
Control	45	360.7 (99.0)	39	366.7 (100.5)	36	372.4 (103.3)	33	367.6 (110.0)
WECT: steady state (VO_2_ in mL/min/kg)								
Intervention	40	14.7 (2.8)	26	16.0 (2.3)	28	15.6 (2.4)	26	16.5 (3.1)
Control	42	14.0 (2.9)	36	14.4 (2.9)	33	14.7 (3.1)	28	15.0 (3.8)

Abbreviations: HDL = high-density lipoprotein; IQR = interquartile range; LDL = low-density lipoprotein; MVPA = moderate-to-vigorous physical activity; MVT = maximal voluntary torque; SF-36 = Short Form 36-Item; VLDL = very low–density lipoprotein; VO_2_ = oxygen consumption; WECT = walking energy consumption test.

Steps, time spent in MVPA, and ACTIVLIM are shown as median [interquartile range]. All other variables are shown as mean (SD).

**Table 4:** Intervention Effects of the Secondary Outcomes at the Different Measurement Occasions

	Over time		After intervention		6-mo follow-up		12-mo follow-up	
	Mean (95% CI)	*p* Value	Mean (95% CI)	*p* Value	Mean (95% CI)	*p* Value	Mean (95% CI)	*p* Value
Steps	242 (−585 to 1,069)	0.564	729 (−330 to 1,789)	0.176	−429 (−1,528 to 670)	0.442	339 (−737 to 1,416)	0.534
Time spent in MVPA	−1.4 (−38.6 to 35.8)	0.940	11.3 (−44.6 to 67.2)	0.689	−11.3 (−66.2 to 43.6)	0.684	7.6 (−49.7 to 65.0)	0.792
SF-36: physical component score	1.1 (−1.7 to 3.8)	0.439	0.7 (−2.7 to 4.1)	0.702	1.5 (−1.9 to 5.0)	0.379	1.0 (−2.5 to 4.5)	0.586
SF-36: mental component score	0.1 (−1.1 to 1.2)	0.910	0.4 (−1.1 to 1.9)	0.586	0.0 (−1.6 to 1.5)	0.969	−0.2 (−1.7 to 1.4)	0.818
ACTIVLIM	0.1 (−0.3 to 0.5)	0.506	0.0 (−0.4 to 0.4)	0.950	0.3 (−0.1 to 0.7)	0.176	0.1 (−0.3 to 0.5)	0.639
Self-efficacy scale	0.5 (−0.8 to 1.7)	0.454	0.3 (−1.2 to 1.9)	0.670	0.4 (−1.2 to 1.9)	0.616	0.7 (−0.8 to 2.3)	0.357
MVT musculus quadriceps strongest leg	−0.3 (−6.6 to 6.1)	0.932	−1.4 (−9.2 to 6.4)	0.724	1.1 (−7.1 to 9.3)	0.792	0.1 (−8.2 to 8.4)	0.988
MVT musculus triceps surae strongest leg	−0.8 (−5.9 to 4.3)	0.760	−0.9 (−7.6 to 5.8)	0.784	0.9 (−6.3 to 8.2)	0.797	−1.9 (−9.3 to 5.4)	0.602
Cholesterol	−0.1 (−0.4 to 0.2)	0.502	−0.2 (−0.5 to 0.2)	0.265	0.0 (−0.3 to 0.4)	0.880	−0.1 (−0.5 to 0.3)	0.558
HDL	0.0 (−0.1 to 0.0)	0.321	0.0 (−0.1 to 0.1)	0.843	0.0 (−0.1 to 0.1)	0.510	−0.1 (−0.2 to 0.0)	0.085
LDL	−0.1 (−0.4 to 0.1)	0.298	−0.2 (−0.6 to 0.1)	0.139	−0.1 (−0.4 to 0.3)	0.728	−0.1 (−0.4 to 0.2)	0.545
VLDL	0.0 (−0.2 to 0.2)	0.873	0.0 (−0.3 to 0.2)	0.777	0.1 (−0.2 to 0.4)	0.451	0.0 (−0.3 to 0.3)	0.991
Triglycerides	0.1 (0.0 to 0.3)	0.094	0.1 (−0.1 to 0.2)	0.559	0.2 (0.0 to 0.4)	0.049	0.1 (0.0 to 0.3)	0.152
Glucose	−0.1 (−0.2 to 0.0)	0.186	0.0 (−0.2 to 0.1)	0.576	0.0 (−0.2 to 0.2)	0.752	−0.2 (−0.4 to −0.1)	0.008
Waist circumference	1.3 (−1.0 to 3.6)	0.258	2.4 (0.1 to 4.7)	0.042	1.5 (−0.7 to 3.8)	0.177	1.3 (−1.0 to 3.6)	0.258
Creatine kinase	−0.7 (−52.0 to 50.6)	0.978	46.3 (−39.0 to 131.5)	0.286	−5.6 (−95.8 to 84.5)	0.902	−49.4 (−140.3 to 41.5)	0.285
Blood pressure: diastolic	1.0 (−2.5 to 4.4)	0.571	1.3 (−2.8 to 5.5)	0.521	0.8 (−3.6 to 5.1)	0.725	0.6 (−3.8 to 5.0)	0.774
Blood pressure: systolic	−0.6 (−5.6 to 4.4)	0.814	0.4 (−5.9 to 6.8)	0.892	−2.7 (−9.5 to 4.0)	0.425	0.7 (−6.2 to 7.5)	0.848
WECT: distance	5.1 (−7.3 to 17.5)	0.411	1.5 (−12.9 to 15.8)	0.840	10.8 (−4.1 to 25.8)	0.153	3.9 (−11.2 to 19.0)	0.611
WECT: steady state	0.9 (0.0 to 1.8)	0.041	1.1 (−0.0 to 2.2)	0.053	0.8 (−0.3 to 1.9)	0.173	0.9 (−0.3 to 2.0)	0.145

Abbreviations: HDL = high-density lipoprotein; LDL = low-density lipoprotein; MVPA = moderate-to-vigorous physical activity; MVT = maximal voluntary torque; SF-36 = Short Form 36-Item; VLDL = very low–density lipoprotein; WECT = walking energy consumption test.

### Adverse Events

Twenty-five adverse events occurred in 20 participants in the control group and 22 adverse events in 16 participants in the intervention group. The reported adverse events were mainly falls (26%), pain (17%), or a COVID-19 infection (15%). Furthermore, there were 3 serious adverse events in the intervention group (stroke, coughing blood, vestibular neuritis) and 2 in the control group (stroke, coronary artery stenosis), all unrelated to the intervention or study protocol. There were no significant differences in creatine kinase levels between both groups (Table 4).

### Classification of Evidence

This study provides Class II evidence that a 6-month personalized aerobic exercise program combined with coaching improves maximal aerobic capacity in patients with NMDs without effective cures.

## Discussion

This multicenter RCT in persons with different NMDs showed that combined personalized home-based aerobic exercise and coaching improved physical fitness (VO_2peak_) by 2.2 mL/min/kg directly after intervention compared with usual care. The positive effects were significant over time in favor of the intervention group, although the intervention effects diminished slightly at the 6-month and 12-month follow-up assessments. There was no evidence of improvement in daily physical activity, quality of life, physical functioning, or markers of metabolic syndrome. The intervention was safe; creatine kinase levels remained unchanged, and adverse events were similar in the intervention and control groups.

The 2.2-mL/min/kg improvement in VO_2peak_ found in this large RCT, in a group with different NMDs, demonstrates the beneficial effects of our combined aerobic exercise and coaching program. Our study findings add substantially to the existing body of evidence for the beneficial effects of aerobic exercise, which was up to now mainly based on low-quality studies that were uncontrolled, lacked intention-to-treat analyses, or were underpowered.^5-7^ Adequate VO_2peak_ levels have a positive impact on various health parameters (including mortality) and reduce the risk of developing noncommunicable diseases, such as cardiovascular disease, osteoporosis, and obesity,^33-35^ to which people with NMD are prone.^1^ The 2.2-mL/min/kg (10%) increase in VO_2peak_ that we found is consistent with that in previous aerobic exercise studies in NMD^5-7^ and other clinical populations such as stroke survivors, cancer survivors, and people with Parkinson disease.^36-38^ Furthermore, the findings that the improvement in VO_2peak_ is greater than the minimal clinically important difference of 1.8 mL/min/kg in NMD^6^ and that 44 of 91 participants (48%) had a baseline VO_2peak_ lower than 20.0 mL/min/kg, the threshold below which independence is known to be impaired in older people,^39^ highlight the clinical relevance of our findings to the population of interest.

As one of the first exercise studies in NMD to focus on the integration of physical activity into daily life and long-term assessments, it is promising that we found improved physical fitness levels over a period of 1 year (on average 1.7 mL/min/kg), although the effect slightly diminished after the intervention. Our intervention combined aerobic exercise with coaching to support the transition from therapist-supervised exercise to continued physical activity integrated into daily life. This coaching program was based on MI, which has been shown to be effective in changing health behaviors in different populations,^40,41^ and physical activity levels in the general adult population^42^ and chronic health conditions.^43^ The combination of coaching and aerobic exercise is likely to have played an important role in maintaining the effects in VO_2peak_ over time. Additional strategies, such as booster coaching sessions^44^ every 3–6 months, could be considered to counteract the diminished long-term effect of the intervention.^45^

Despite the improved physical fitness, we did not find significant improvements in physical functioning, daily physical activity, quality of life, or markers of metabolic syndrome. While some previous exercise studies in NMD found improvements in these (secondary) outcomes, recent systematic reviews reported that most studies, like ours, did not.^5-7^ There are several possible explanations. The high variability in some of the secondary outcomes likely required a larger sample size to ascertain a significant effect. For example, daily physical activity in the intervention group was estimated to be 729 steps higher than in the control group, but with a high outcome variability (Table 4), and, therefore, not significant. Other explanations include that the selected secondary outcomes were not specific enough to capture improvements, or that the combination of aerobic exercise and coaching is not sufficient to improve physical functioning and quality of life. Both the ACTIVLIM and SF-36 questionnaires include items that may not necessarily change with improvements in VO_2peak_, such as bathing, dressing, or carrying loads or groceries. This may result in a lack of improvements in the overall scores. Alternative outcomes to consider are the Goal Attainment Scale and the Canadian Occupational Performance Measure, which aim to identify and prioritize personal goals for meaningful daily activities and have been used in previous studies in NMD.^9,46^ As suggested by others,^6,10^ the addition of more task-specific exercises, such as walking or balance, may be necessary for effects on physical functioning and quality of life. Furthermore, outcomes such as metabolic syndrome markers may require longer durations to exhibit significant changes. Further research should explore the association between changes in VO_2peak_ and changes in functional outcomes, which may better reflect potential improvements in daily life.

Unlike most exercise studies in NMD, which have used hospital-based aerobic exercise programs, we deliberately designed a home-based polarized aerobic exercise program because it is easier to implement in daily life. The positive effects that we found, including the limited adverse events associated with our program and the lack of increase in creatine kinase levels, demonstrate that it is possible for people with NMD to safely improve physical fitness at home. Although our intervention included several on-site sessions as part of the aerobic exercise and coaching program, hospital-based exercise programs are generally more burdensome regarding travel time and therapist support. Despite less intensive supervision, the results of our home-based program, including adherence rates, are similar to those of hospital-based exercise programs.^5,6^ Components that contributed to these promising results include the use of a personalized and polarized aerobic exercise program and the use of the ReVi app. While it is not possible to disentangle the contribution of these different components, the ReVi app likely played an important role, as we know from a previous pilot study that the app motivates people with NMD to complete their program and helps them to stay within their target intensity zones.^18^ Our findings support the transition to home-based exercise programs for people with NMD, which is especially important given the current shortage of physical therapy staff and reductions in health care services worldwide.^47^

Our trial has several limitations. We purposefully included people with different NMDs, because we hypothesized that common mechanisms underlie reduced physical fitness in different types of NMD. This approach, which was similar to a study on a self-management program in NMD,^9^ increased the generalizability of our results to the population with NMD at large, but at the expense of heterogeneity and limited possibilities for NMD subgroup analyses. In this respect, it was reassuring that groups were well balanced regarding clinical characteristics (e.g., type of NMD, MMT, walking aids, and functional ambulation levels). Furthermore, determining a “reduced physical fitness” is challenging. Despite discussing fitness and activity levels during screening, some participants had a rather high VO_2peak_ at baseline. This may have led to an underestimation of the intervention effects as the effects seem to be more pronounced in people with a lower baseline VO_2peak_.^5^ Moreover, almost all participants were ambulant and of relatively older age, potentially having more comorbidities, limiting generalizability to other, for instance, ambulant and younger populations. The COVID-19 pandemic also affected our study. More than 20 assessments could not be performed because of the COVID-19 measures, which had a major impact on the study completion rate of 73% (Figure). The COVID-19 measures changed physical activity behavior of people with NMD,^48,49^ and the closure of sports facilities and physiotherapy practices is assumed to have had a larger impact on participants in the intervention group because some of them relied on these facilities for their exercise program or sports activities. It likely also affected certain outcomes, such as exercise self-efficacy, and consequently, it may have led to an underestimation of the intervention's effectiveness.

In conclusion, this study demonstrates that combined personalized home-based aerobic exercise and coaching is safe and improves physical fitness in people with various NMDs, but without evidence of improved daily physical activity, quality of life, physical functioning, and metabolic syndrome markers. The home-based approach of our program has good potential for a wider implementation in clinical practice. Future research should explore the association between changes in VO_2peak_ and changes in functional outcomes, which may better reflect potential improvements in daily life, and should evaluate additional strategies to support the transition to continued physical activity integrated into daily life, and therewith counteract the diminishing long-term effect of the intervention.

## Glossary

ACSM: American College of Sports Medicine
AMC: Academic Medical Center
CMT: Charcot-Marie-Tooth disease
COVID-19: coronavirus disease 2019
CPET: cardiopulmonary exercise test
I'M FINE: IMproving FItness in NEuromuscular diseases
IQR: interquartile range
MI: motivational interviewing
NMD: neuromuscular disease
PPS: post-polio syndrome
RCT: randomized controlled trial
SF-36: Short Form 36-Item
VO_2peak_: peak oxygen uptake
VT1: first ventilatory threshold
